# Temporal Code-Driven Stimulation: Definition and Application to Electric Fish Signaling

**DOI:** 10.3389/fninf.2016.00041

**Published:** 2016-10-06

**Authors:** Angel Lareo, Caroline G. Forlim, Reynaldo D. Pinto, Pablo Varona, Francisco de Borja Rodriguez

**Affiliations:** ^1^Grupo de Neurocomputación Biológica, Departamento de Ingeniería Informática, Escuela Politécnica superior, Universidad Autónoma de MadridMadrid, Spain; ^2^Department of Data Analysis, Faculty of Psychology and Educational Sciences, Ghent UniversityGhent, Belgium; ^3^Laboratory of Neurodynamics/Neurobiophysics, Department of Physics and Interdisciplinary Sciences, Institute of Physics of São Carlos, Universidade de São PauloSão Paulo, Brazil

**Keywords:** computational biology, signal processing, information theory, electric fish, neuroinformatics

## Abstract

Closed-loop activity-dependent stimulation is a powerful methodology to assess information processing in biological systems. In this context, the development of novel protocols, their implementation in bioinformatics toolboxes and their application to different description levels open up a wide range of possibilities in the study of biological systems. We developed a methodology for studying biological signals representing them as temporal sequences of binary events. A specific sequence of these events (code) is chosen to deliver a predefined stimulation in a closed-loop manner. The response to this code-driven stimulation can be used to characterize the system. This methodology was implemented in a real time toolbox and tested in the context of electric fish signaling. We show that while there are codes that evoke a response that cannot be distinguished from a control recording without stimulation, other codes evoke a characteristic distinct response. We also compare the code-driven response to open-loop stimulation. The discussed experiments validate the proposed methodology and the software toolbox.

## 1. Introduction

Most biological systems operate in a history-dependent manner, thus feedback is essential for their operation. For years scientists have studied biological systems probing them in an unidirectional stimuli-response manner. Recently, open-loop stimulation is being complemented with closed-loop approaches to stimulate biological systems depending on their ongoing activity (Potter et al., 2014; Roth et al., 2014).

In closed-loop stimulation the activity of the biological signal is monitored through specific probes and an event detection algorithm is used to drive the stimulation protocol through an actuator. The stimulation can be modified depending on the feedback received from the system. It can be delivered based on different events, or sequences of events, detected from signals or behavior produced by the system.

Closed-loop stimulation has been successfully implemented in neurophysiology under the concept of dynamic clamp (Robinson and Kawai, 1993; Sharp et al., 1993; Destexhe and Bal, 2009) Activity-dependent protocols have been used to address neuromuscular control (Sponberg et al., 2011), and are commonly employed in modern EEG, ECoG, and fMRI studies in humans, e.g., (Schalk and Leuthardt, 2011; Fernandez-Vargas et al., 2013; Ruiz et al., 2014) and in other fields like neuroethology (Muniz et al., 2011; Chamorro et al., 2012; Madhav et al., 2013; Roth et al., 2014).

These closed-loop technologies call for new perspectives in designing stimulus-response protocols. In this context, a relevant approach is the adaptive sampling, a model-driven stimulus exploration which has been effectively applied to sensory physiology research (Benda et al., 2007). This methodology uses an information-theoretic analysis (Paninski, 2005; Lewi et al., 2009) to maximize the information about the system. Input stimulus and outputs are analyzed to dynamically select the stimulus and reduces the uncertainty about the system (Lewi et al., 2009; DiMattina and Zhang, 2011, 2014). This approach improves the quality of the experimental data obtained and allows for better discrimination between hypotheses.

The generalization of closed-loop techniques opens a wide variety of possibilities to deliver stimuli in an activity-dependent manner to monitor and study biological systems (Chamorro et al., 2012). Most closed-loop techniques discriminate between the presence or absence of a certain event or use the instantaneous value of a monitored signal to trigger the stimulus (Muniz et al., 2011; Berényi et al., 2012; Ehrens et al., 2015; Forlim et al., 2015). However, it is known that neural systems can encode relevant information in a sequential manner, which gives rise to complex temporal patterns (e.g., Laurent et al., 1996; Nádasdy, 2000; Rodriguez and Huerta, 2004). For example the temporal coding scheme claims that some neural systems encode information in the precise temporal structure of the spike train (e.g., Dayan and Abbott, 2003; Brochini et al., 2011).

There is an inherent difficulty to analyze temporal coding in biological systems because the distribution of events allows a wide variety of codes. Additionally some of these codes may overlap. Closed-loop approaches can be used to overcome this problem. A code can be studied when its defining sequence is used to trigger the stimulation and distinctly conditions the response of the system.

In this paper we introduce temporal code-driven stimulation: a methodology to study temporal patterns of events in biological systems using binary digitization of analog biological signals. This methodology represents event trains as binary codes. A code is selected to be used as a trigger for stimulation. Accordingly, the biological activity determines the stimulation timing. The system's response to this closed-loop can be studied to assess sequential processing in a wide variety of experimental setups.

A well-known example of a biological system with temporal coding and easy to measure pattern activity is weakly electric fish (Bullock et al., 2006; Baker et al., 2013). These animals are a convenient model to analyze information processing and communication mechanisms due to their remarkable electrosensory system. In active electroreception, the animal generates its own electric fields with an electric organ located at the tail (Caputi et al., 2002; Bullock et al., 2006; von der Emde et al., 2010). Electric organ discharges (EODs) are then detected as distortions in the electric field around the fish body using electroreceptors, and this signal can be easily detected non-invasively. It can be done in alive and active freely-behaving animals using the appropriate hardware (Jun et al., 2012; Forlim and Pinto, 2014; Forlim et al., 2015).

For our validation experiments, we used fish of the species *Gnathonemus Petersii* which generate electric pulses. The pulse waveform of this fish is stereotyped, but the time between discharges vary considerably (Carlson, 2002; Baker et al., 2013; Carlson and Gallant, 2013; Forlim and Pinto, 2014). The inter-pulse intervals (IPIs) carry information about the behavioral state of the fish (Carlson, 2002; Carlson and Gallant, 2013; Forlim and Pinto, 2014), i.e., information is encoded in the temporal structure of these IPI patterns. There is a link between discharge patterns and submissive, aggressive and exploratory behavior (Carlson, 2002). For example, shortening of IPIs has been observed during chasing and attacking behavior (Bell et al., 1974; Kramer and Bauer, 1976). Furthermore, these changes are also correlated with different swimming patterns and motor behavior (Kramer, 1996; Forlim and Pinto, 2014). Finally, IPIs also change depending on external electrical stimulation (Kramer, 1979; Forlim et al., 2015).

We have developed a toolbox as a module for a real-time platform (Muñiz et al., 2005; Muñiz et al., 2008; Muñiz et al., 2009) to implement temporal code-driven stimulation. As an illustrative proof of concept for our methodology, we report here on temporal code-driven stimulation in the context of electric fish signaling.

This paper is organized as follows: Section 2 explains the approach to define temporal code-driven stimulation. Section 3 details the methods used to implement and test temporal code-driven stimulation. Preliminary results obtained applying temporal code-driven stimulation to the study of electric fish signaling are presented in Section 4. Finally, we discuss the results and issues related with the application of this methodology in Section 5.

## 2. Approach

In this section we describe in a general manner the concept of temporal code-driven stimulation, an activity-dependent stimulation triggered by specific codes. We follow a black-box experimental approach, studying a system in terms of its response to stimulus delivered by specific sequences present in its activity. We define a code as a predetermined temporal sequence of events emitted by the system. These activity patterns are evaluated studying changes in the system's activity under code-driven stimulation. Then, we compare these changes obtained to those that occur under other stimulation conditions.

**Figure 1:**
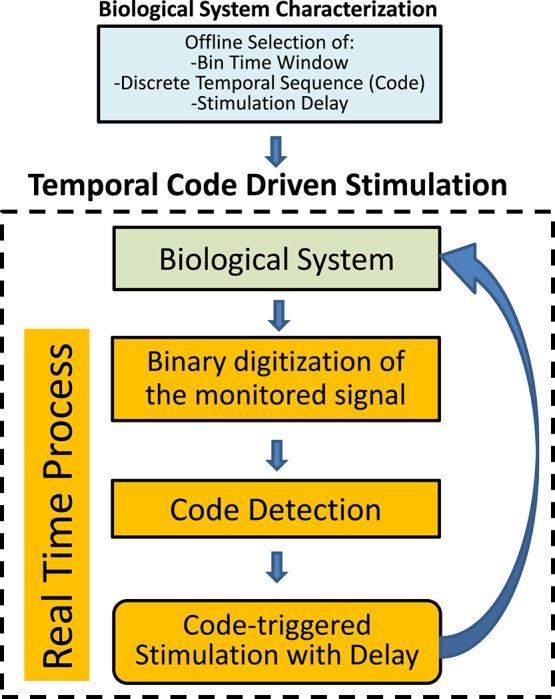
**Schematic representation of the closed-loop temporal code-driven stimulation**. Preselection of the parameters is based on previous offline analysis of the system. The biological system signal is acquired, digitized to a binary sequence and words are detected in order to guide the stimulation function.

The closed-loop stimulation protocol is depicted in Figure 1. The system is first characterized in order to select the parameters for binary digitization, word length and stimulation delay. The probability of occurrence of each code is obtained as a function of these parameters. We use the information theory approach for parameter selection, as explained in Section 3.

Once the parameters are selected, the closed-loop starts (Figure 1). First, the biological signal is acquired. The signal is digitized to a binary sequence considering the presence or absence of a predefined event. Temporal sequences of events are detected in real-time as binary codes (words). The stimulation is delivered, with a predetermined delay, after the detection of a pattern that matches the preselected word.

By delivering the stimulation as a function of the system's activity we can analyze the integration of temporal sequences of events in a closed-loop fashion and the influence of different patterns in the associated information processing.

It is highly relevant to compare the results of this method with those obtained with an open-loop stimulation protocol, where stimulation does not depend directly on the system's activity. Thus, we also designed an open-loop protocol where stimulation is randomly delivered, but on average as frequently as in the closed-loop.

## 3. Methods

**Figure 2:**
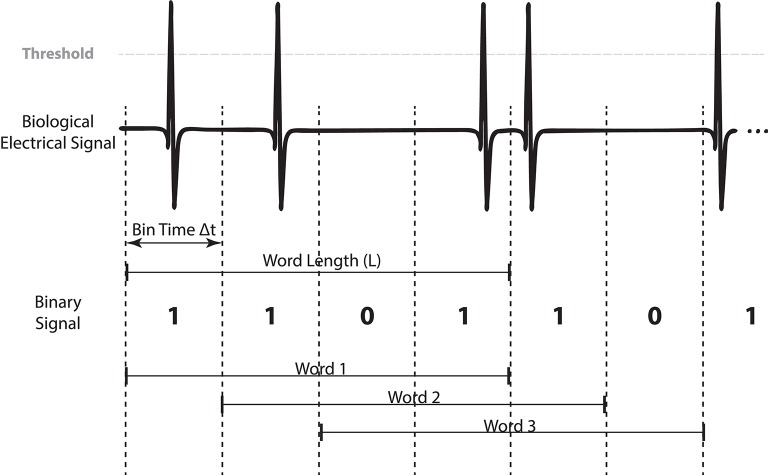
**Binary digitization and word formation example (4 bits) from a generic biological signal where pulse events are detected**. Bits are superimposed between words as we use a shift of 1 bit in order to get all possible words resulting from the signal.

**Figure 3:**
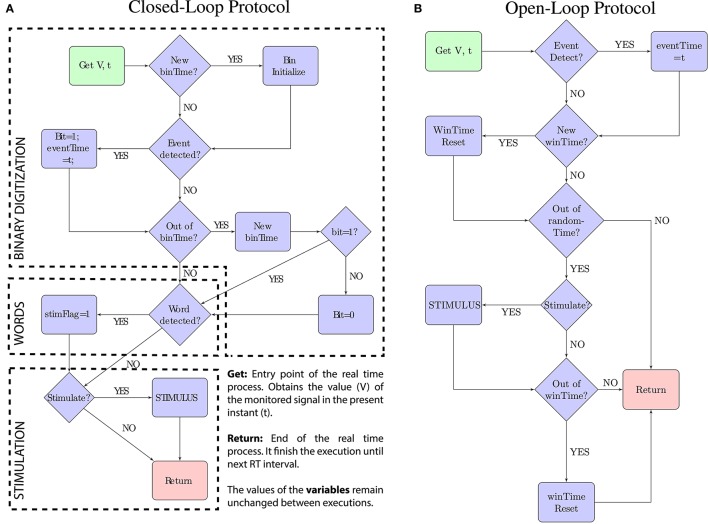
**Schematic representations of the real-time protocols**. The code-driven closed-loop protocol is described by the scheme **(A)**. The open-loop stimulation protocol used as a comparison is described by the scheme **(B)**. In both, the green box represents the entry point and the red box represents the exit point. Each function is executed on every real-time interval (time = *t*) and starts acquiring the value of the monitored signal (*V*). The trigger “*Stimulate?*” in both protocols depends on the pulse-stimulus delay (fixed value or range) introduced as a parameter to the protocol.

Our methodology requires a previous characterization of the biological signal either offline or online (Section 3.1). In this first step, the parameters for binary digitization are selected, and then, words are formed (Figure 2). In Section 3.2 we describe the functional requirements of the toolbox, the specific functionality that our toolbox has to accomplish, including its real-time constrains. In Section 3.3 we describe the stimulation protocols and the way they operate (summarized in Figure 3). Finally, we apply our toolbox to the study of electric signaling in weakly electric fish (Section 3.4).

Our code-driven closed-loop interaction with biological systems depends on the performance of our methods. Temporal constraints are very strict, as biological systems are very sensitive to small variations in the stimulation timing.

We implemented our methods using general purpose computers with a real time operating system (RTOS) for better re-usability and portability. We chose Real Time Application Interface, RTAI (https://www.rtai.org/), a real time linux kernel extension. RTAI provides an application programming interface (API) which permits the execution of periodic tasks in hard real time. Thus, it assures preemption in every function related with biological system interactions.

### 3.1. Signal analysis and processing

To digitize the monitored signal to a binary sequence, we divide it into *N* time windows of size Δ*t*. Then we assign a bit value depending if an event is detected in the time window: 1 when an event is present and 0 otherwise. This results in a discrete temporal sequence {*e*_*t*_; *t* = 1…*N*} where *e*_*t*_ could be 0 or 1.

The system's activity is mapped to a consecutive set of bits that we call word (Figure 2). For a given Δ*t* we define a word $W_{t}^{L}$ of size L (number of bits) at time *t* as a sequence of symbols $W_{t}^{L}=\left\{e_{t\;-\;L};\ldots ;e_{t\;-\;1};e_{t}\right\}$, where *t* is the time when the sequence is detected. Thus, $W_{t}^{L}$ is the sequence that happened at time *t* formed by the events between *t* − *L* and *t*. Accordingly, there are *N* − *L* + *1* words of *L* bits in each time series (Figure 2).

In order to digitize to a binary sequence and obtain the words, Δ*t* and L must be selected. If Δ*t* is too small, there will be many bins without pulses, therefore set as zero. On the other hand, if Δ*t* is too large, there will be pulses in all bins, that is, all bits will be set to 1. To overcome this issue, we choose the bin time Δ*t* that maximizes the entropy of the binary signal (Jaynes, 1957).

For a given set of words $W^{L}=\left\{w_{1}^{L},w_{2}^{L},\ldots ,w_{n}^{L}\right\}$, the entropy is calculated as:

$$H\left(W^{L}\right)=-\underset{i}{\sum }P\left(w_{i}^{L}\right)logP\left(w_{i}^{L}\right)$$

where $P\left(w_{i}^{L}\right)$ is the probability of occurrence of $w_{i}^{L}$. The entropy *H*(*W*^*L*^) is related to the variability of the set and it represents the signal capability of encoding information. The entropy is computed using several values of Δ*t* and L.

The practical application of this method to neural coding and spike train analysis is prone to a systematic error in the stimation of entropy, as a consequence of the limited number of samples in experimental data (Panzeri et al., 2007). This bias can be stimated as:

$$BIAS\left(H\left(W^{L}\right)\right)=-1/2Nln\left(2\right)\left[\bar{W^{L}}-1\right]$$

where $\bar{W^{L}}$ is the number of relevant words and *N* the number of samples, or different words in the experimental data. This BIAS can be used to correct the entropy value.

### 3.2. Software toolbox requirements

We divide the functional requirements of the toolbox in three different procedures (detailed in Table 1):

- F1. Signal analysis to obtain the binary word distribution.
- F2. Stimulation protocol that operates in closed-loop, detecting a preselected binary word and firing the stimulation (with a preselected delay).
- F3. Open-loop protocol.

**Table 1:** **Functional requirements for the toolbox**.

**Code**	**Description**	**Operation mode**
F1	Binary words distribution over time	Offline/RT
F1.1	Bin time selection	Offline/RT
F1.2	Word-length selection	Offline/RT
F1.3	Signal binary digitization	Offline/RT
F1.3.1	Event detection	Offline/RT
F1.4	Histogram generation	Offline/RT
F2	Closed-loop stimulation protocol	RT
F2.1	Bin-time selection	Offline
F2.2	Word selection	Offline
F2.3	Signal Binary Digitization	RT
F2.3.1	Event detection	RT
F2.4	Binary word detection	RT
F2.5	Fixed-delay stimulation	RT
F3	Open-loop protocol	RT
F3.1	Stimulation time period selection	Offline
F3.2	Fixed-delay stimulation	RT
F3.2.1	Event detection	RT

We divide this requirements in three different procedures (F1, F2, and F3). Requirement F1 corresponds to the analysis of signals, which can be performed online or offline in pre-recorded signals. Requirement F2, closed-loop stimulation and F3, open-loop stimulation, are online protocols. The operation mode column describes two modes of operation: Offline and RT (Real time, online with strict time constraints).

The column tagged as “*Operation mode”* in Table 1 presents the temporal requirements for the different functions:

1. It operates while the signal is being acquired (online manner in real-time, F1, F2, and F3)
2. It analyzes a pre-recorded signal (offline manner, F1).

Function implementing F1 calculates the probability of occurrence of the distinct binary words. The offline version generates histograms of the words in a pre-recorded signal for different Δ*t* and *L* parameters. It also calculates the Shannon entropy, the bias and the corrected entropy. This is a basic characterization tool in order to select appropriate parameter values. Nevertheless, other software tools for information-theoretic analysis of neural data can be used (Ince et al., 2010) independently or in conjunction with our tool. For instance, some of these tools are PyEntropy^1^, R Entropy^2^, or MATLAB Spike Train Analysis Toolbox^3^. In addition, the online version shows instant (in a given time window) and accumulated histograms of words of the ongoing activity of the system. The functional requirements for this program are listed in Table 1 tagged with code F1.

The online functions provided in our toolbox require the presence of a hardware-software platform to collect the signal emitted by the biological system. This platform must be able to generate stimuli and present them to the biological system in real time.

### 3.3. Stimulation protocols

There are two different real-time stimulation protocols in the toolbox: a closed-loop protocol and a open-loop one (functionalities F2 and F3 in Table 1, Figure 3).

The closed-loop protocol stimulates the system triggered by a preselected binary word. It operates in real time and thus it has to keep track of the digitization window, the current bin and the previous bits among executions. In our experiments we chose 17 kHz as a safe sampling rate to correctly detect the events and accomplish the time constraints. Parameters (Δ*t* and trigger binary word) must be previously selected in an offline manner.

The implementation of these protocols is flexible and generalizable to different applications. The binarization time, the word length and the trigger code are parameters that are selected for each experimental model. The stimulation is triggered through a generic interface. This interface must be specifically adapted for each biological system, i.e., to describe the specific code and stimulus. This makes our implementation independent from a specific system's characterization and from the stimulation function (see Supplementary Material for more details about real-time implementation of these protocols).

The functional requirements for this code-driven stimulation protocol are listed in Table 1 tagged with code F2.

The open-loop protocol allows us to compare changes in the system due to stimulation triggered by previous activity of the fish (closed-loop) with stimulation that does not take into account fish's activity (open-loop). It divides the temporal sequence into time windows of the same duration and triggers one stimulus per window, randomizing inside this window the precise time when the stimulus is sent. It also operates in real time so it has to keep track of the window and the random stimulation parameters among executions.

The functional requirements for this open-loop protocol are listed in Table 1 tagged with code F3.

#### 3.3.1. Closed-loop protocol

The closed-loop protocol (Figure 3A) monitors the signal value (V) and the precise time (*t*) when the real-time task takes place. The protocol executes this real-time task 17 K times per second and performs three processes: (1) the binary digitization, (2) the word detection, and (3) the stimulation:

The binary digitization process (1) implements the online windowing and thresholding protocol. The signal is windowed in intervals of *binTime* duration. If an event is detected (*V* > threshold) during this interval, the corresponding bit is set to 1. Otherwise, the bit is set to 0. The exact time when the event is detected is stored (*eventTime*) and used for the stimulation.

The word detection process (2) checks coincidences between the trigger word and the word formed after setting the last bit. If both match, the stimulation flag is activated: *stimFlag* = 1.

Finally, the stimulation process (3) calculates the stimulation delay and delivers the stimulus in the precise time *t* = *eventTime* + *delay*).

#### 3.3.2. Open-loop protocol

The open-loop protocol (Figure 3B) delivers stimulation at the same average frequency of that in the code-driven sessions. To achieve this, the open-loop stimulation windows the signal in intervals of (*winTime*) duration and triggers one stimulus per window. The stimulus is randomly triggered anytime during that window to avoid regularity.

In this protocol *winTime* is selected to achieve the same average stimulation frequency as in code-driven sessions. This interval can be calculated after the the code-driven stimulation dividing the number of stimuli delivered during that session by the total time of the session.

To accomplish randomly triggered stimulation within each time window, a random time (*randomTime* < *winTime*) is selected at the beginning of each window. The process waits for this random time before activating the stimulation flag.

The process does not immediately stimulate after waiting the *randomTime* period, as it also has to accomplish the pulse-stimulus delay restrictions. This delay is also preselected to perform stimulation with the same delay than in code-driven stimulation.

In summary, *V* is monitored and once an event is detected, the precise moment in which the pulse takes place (*t*) is stored (*eventTime* = *t*). If the stimulation flag is active, the process waits for the preselected delay and then, the stimulation is delivered at the instant when *t* = *eventTime* + *delay*.

It is important to note that in the open-loop protocol the stimulation is not triggered by the activity of the biological system (Table 2). However, both open-loop and closed-loop stimulation sessions use the same stimulus type, pulse number, and delay.

**Table 2:** **Comparison of closed-loop and open-loop protocols**.

**Closed-Loop Protocol**	**Open-Loop Protocol**
Temporal code-driven stimulation	Periodic-triggered stimulation
Selected code as trigger	Random code as trigger
Same number of stimuli	
Same stimuli	
Same pulse-stimulus delay	

### 3.4. Electric fish signaling case

Electrical signaling is used by weakly electric fish to communicate with other individuals, to locate objects, food, and also to navigate (von der Emde, G. 1999). *Gnathonemus Petersii*, the species that we used, emmit a pulse-type signal, where pulses are very fast voltage transients in the order of milliseconds, and encode information in temporal patterns (Crawford, 1991; Carlson, 2002; Baker et al., 2013).

Real time was crucial for the performance of the toolbox applied to electric fish signaling. Pulse events takes on average 0.8 ms, so in order to establish an effective closed-loop without degrading the quality of service, these events must be detected, processed and a decision made whether sending a stimulus in < 1 ms. The software must respond within strict time constraints, as precise timing in delivering stimulation is essential to study the biological dynamics of the fish (Forlim et al., 2015).

To achieve that, we implemented our toolbox within a generic real-time platform that runs under RTAI (see Supplementary Material). Our real-time protocols were implemented as a module programmed in ANSI C programming language for the RTBiomanager, an open-source real-time platform (Muñiz et al., 2005; Muñiz et al., 2008; Muñiz et al., 2009) that acquired, processed and recorded the signal. The protocols implemented by this module can be used with any other real-time platform that can get signal value with certain frequency and which can generate stimuli triggered from an interface. These protocols are described in Section 3.3.

**Figure 4:**
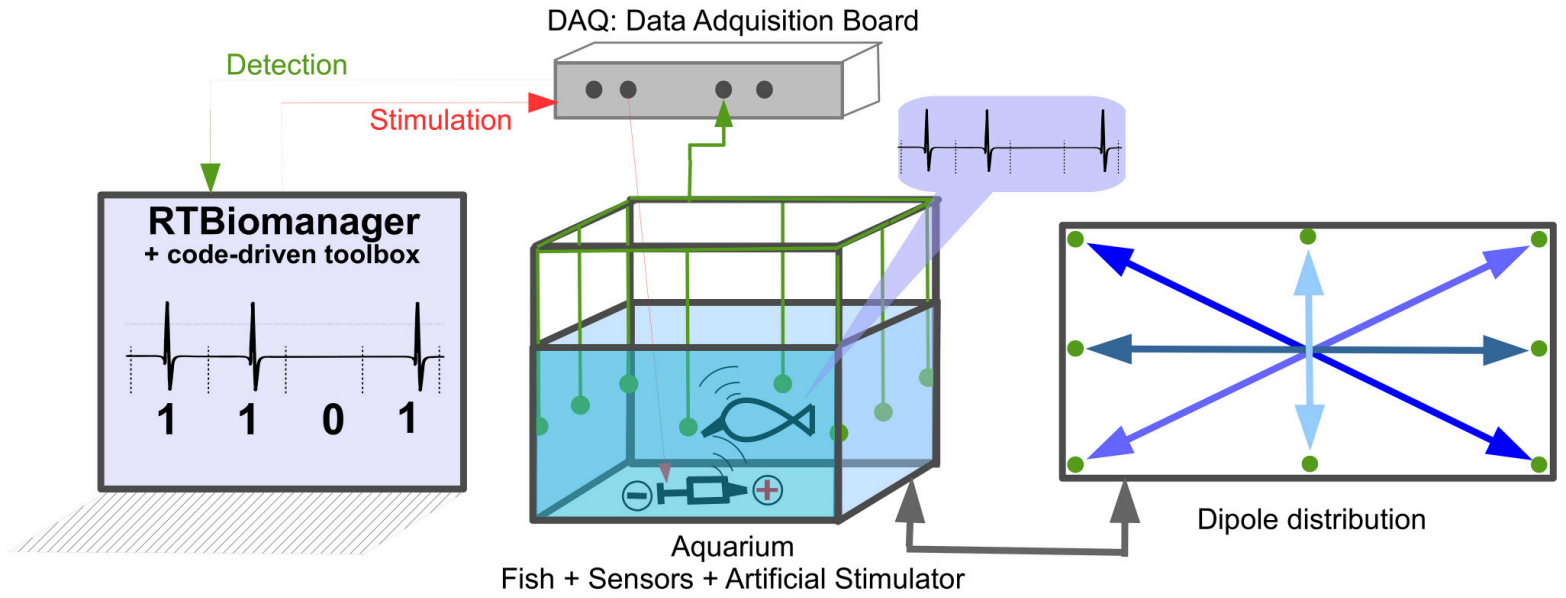
**Setup used for temporal code-driven stimulation in electric fish signaling experiments**. The EODs were measured using four differential dipoles placed at medium depth in the tank, displayed forming an asterisk (see dipole distribution). The grounding electrode is also located in the aquarium. The signal from the dipoles was amplified (TL082 JFET-Input Dual Operational Amplifier with a gain approximately equal to: 91*kΩ*/2.2*kΩ* ≈ 42), summed (LM741 Operational Amplifiers), squared(using AD633 Analog Multiplier) and then digitized at 17 Khz by a DAQ board (NI PCI-6251, National Instruments Corporation). Stimulation is generated by the same board and controlled in real time by software.

The hardware platform (Figure 4) consisted of an aquarium with four differential dipoles to measure the fish signal. The signal received by the dipoles was amplified, summed, and squared. The squared signal was then acquired at 17 kHz by a data acquisition (DAQ) board (NI PCI-6251, National Instruments Corporation) in a PC-compatible computer. To deliver the stimuli, a silver tip dipole was placed at the bottom-middle of the tank. The signal was delivered by the same DAQ Board used to acquire the electrical signal of the fish (for detailed information see Forlim et al., 2015).

**Figure 5:**
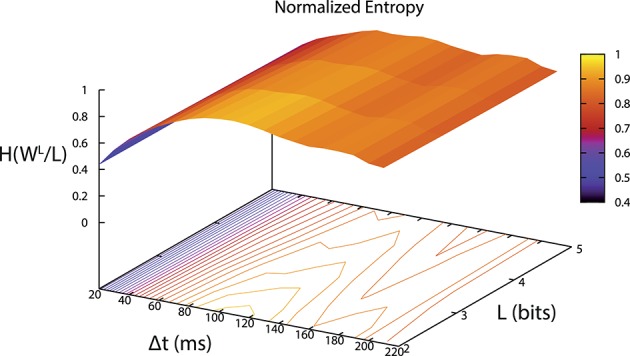
**Entropy per bit of words distribution depending on the parameter values (Δ*t*, L)**. We selected the parameters using a maximum entropy criterion. In this case Δ*t* between 100 and 120 ms and *L* = 2.

The binary digitization process was performed by our software and depended on two parameters (Δ*t*, L). Δ*t* was selected using the maximum entropy criterion (Jaynes, 1957) after an offline analysis of the signal. Entropy was calculated for different values of Δ*t* and L. An example of the resulting entropies is depicted in Figure 5. In this case, Δ*t* that maximized entropy was between 100 and 120 ms.

The electrical stimulus was also controlled by the software. The signal was sent to the aquarium through a 12 cm long silver tip dipole. We chose a 500 Hz sinusoidal stimulus, 2.5 V in amplitude. The stimulus lasted between 200 and 300 ms after the onset. Sine-wave stimuli have been widely used in electrocommunication research (Von der Emde and Bleckmann, 1997; Caputi et al., 1998; Engelmann et al., 2010; Chamorro et al., 2012). It was selected in our case due to its capacity to produce changes in the system (Chamorro et al., 2012) and to avoid overlapping with the fish signal.

The selection of parameters for the closed-loop and the open-loop protocols was different. To set the closed-loop parameters (Δ*t* and preselected trigger), first a control session was performed, then the signal was analyzed offline where Δ*t* was selected using the maximum entropy criterion. The number of bits *L* was an arbitrary choice that should be based on the biological relevance, for our specific case we chose *L* between 2 and 4: 2 bit word is the minimum word which represent a sequential pattern (i.e., that differs from simple event detection) and 4 comprises 16 different words, which we considered adequate to test the protocols.

In our case, we chose a word with high-mean probability of occurrence to ensure good statistics. Low probability words can carry plenty of information, but an insignificant number of stimulations can result in no changes in the system. Hence, in our example we use words with mean probability of occurrence as a compromise between the information they provide and the number of stimulations they will trigger.

On the other hand, for selecting open-loop parameters, the offline analysis was done using the signal from the closed-loop session. We proceeded in this way to ensure similar number of stimuli delivered in both protocols. For that, we divided the running time of the closed-loop session by the number of stimuli delivered, obtaining the mean period between stimuli. The resulting value was used as the time window of the open-loop protocol.

To compare the response to the closed-loop and the open-loop protocols, we used the same stimuli and delay, i.e., the same sinusoidal waveform, 500 Hz sinusoidal shaped, 2.5 V in amplitude.

We analyzed how both protocols changed the fish's electrical behavior by comparing the IPI distributions. IPIs encode information about the behavioral state of the fish (Carlson, 2002; Baker et al., 2013; Forlim and Pinto, 2014). Additionally, to quantify the changes in the IPI distributions between different sessions we used quantile-quantile plots (qqplots) with the same number of samples, discarding IPIs in case of larger distributions (Cleveland, 1993). In this plot, the data from 2 distributions, *X* = {*X*_*i*_} and *Y* = {*Y*_*i*_}, are sorted and plotted against each other. In our case, each *X*_*i*_ (or *Y*_*i*_) represented a IPI from session X (or Y). If IPIs from different sessions comes from similar distribution, the qqplot will show points lying on to the reference line (i.e., *X* = *Y*). This means that changes in the electrical activity are minimal between sessions. If the points lie above the reference line, IPIs discharged in the Y session are larger than those for the X session. Conversely, if points lie below the reference line, IPIs discharged during the Y session are shorter than those for the X session.

## 4. Results

We present preliminary results in the context of the electric fish signaling as a validation of our toolbox. We conducted three different experiments:

1. Code-driven stimulation with minimal codes. In this first set of experiments we addressed the analysis of the fish's response to minimal code-driven stimulation (minimum words that represent a sequential pattern, i.e., that differs from simple event detection) comparing it to the response obtained with pulse-event-triggered stimulation.
2. Code-driven stimulation with longer codes (4 bit words). We analyzed the fish's response to code-driven stimulation using different 4-bit words.
3. Code-driven stimulation vs. open-loop stimulation. We studied the effect of temporal code-driven stimulation vs. open-loop stimulation.

### 4.1. Code-driven stimulation vs. pulse-event triggered stimulation

We defined pulse-driven stimulation as the one that took place after every simple pulse, a code-driven stimulation with word 1. We compared it to stimulation triggered by a word with *L* = 2.

With L=2, the code set was $W_{t}^{2}=\left\{01,11\right\}$ as we chose only words ended by 1 in order to control the pulse-stimulus delay. We chose Δ*t* = 60 ms, that maximized the entropy of the binary signal. The pulse-stimulus delay was set to 10 ms, as it has been shown that pulse-stimulus delays under 100 ms evoks more changes in the electrical behavior (Forlim et al., 2015).

**Figure 6:**
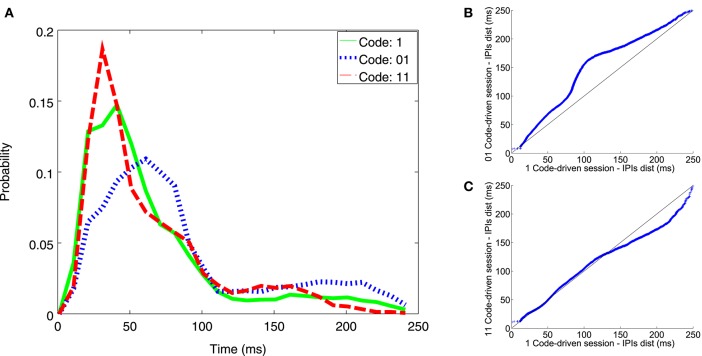
**Illustrative example of IPI histogram and qqplots resulting from experiments addressing temporal coding influence with minimal codes**. The IPI histogram **(A)** represents the distribution of IPIs discharged during code-driven stimulation sessions with different words triggering the stimulation (1 solid line; 01 dashed line; 11 dotted line). IPIs, represented on the X axis, were in the range between 0 and 250 ms and the probability, represented in the Y axis, was normalized. The qqplots represented IPI distributions during pulse event (1) stimulation sessions vs. 2-bit code-driven stimulation sessions (01 **B**; 11 **C**). X = Y is the reference line. In the qqplot that represented IPIs during the stimulation session using pulses vs. IPIs during the 01 stimulation session **(B)** most of the points were above the reference line, thus indicating that IPIs discharged during the 01-session were larger than those from the 1-session. In the qqplot that represents IPIs during stimulation session using pulses vs. IPIs during 11 stimulation session **(C)**, most of the points were below the reference black line, thus indicating that IPIs discharged during the 11-session were shorter than the 1-session, particularly in the range between 160 and 240 ms.

The code-driven closed-loop protocol evoked more changes in the electrical behavior as compared to the pulse event one: the fish fired shorter IPIs (between 20 and 45 ms) in response to word 11 (Figure 6A-dotted line) than those in response to pulse stimuli, indicated here as 1 (Figure 6A-solid line). On the other hand, the fish fired larger IPIs when stimulation was triggered by word 01 (Figure 6A-dashed line) as compared to pulse stimuli 1 (for IPIs between 65 and 90 ms and between 160 and 240 ms).

In the qqplots, one can observe that the points for pulse stimuli 1 vs. word 01 were above the refence line (Figure 6B-black line), indicating that 01 word stimulation increased the probability of firing larger IPIs. The opposite was observed for pulse stimuli 1 vs. word 11 (Figure 6C), specially for IPIs larger than 140 ms. That is, word 11 increased the probability of firing shorter IPIs. See an additional example in Figure S1.

### 4.2. Code-driven closed-loop stimulation with 4 bit words

Here we investigated how the fish responded to different 4 bit words. We selected *L* = 4, so there were 8 different words to trigger the stimulus (2^4^ = 16 possible codes, 8 ended by 1). From the entropy maximization we chose Δ*t* = 80 ms. We selected words with mean probability of occurrence and ended by 1. The words were calculated from the control session before the open-loop protocol.

**Figure 7:**
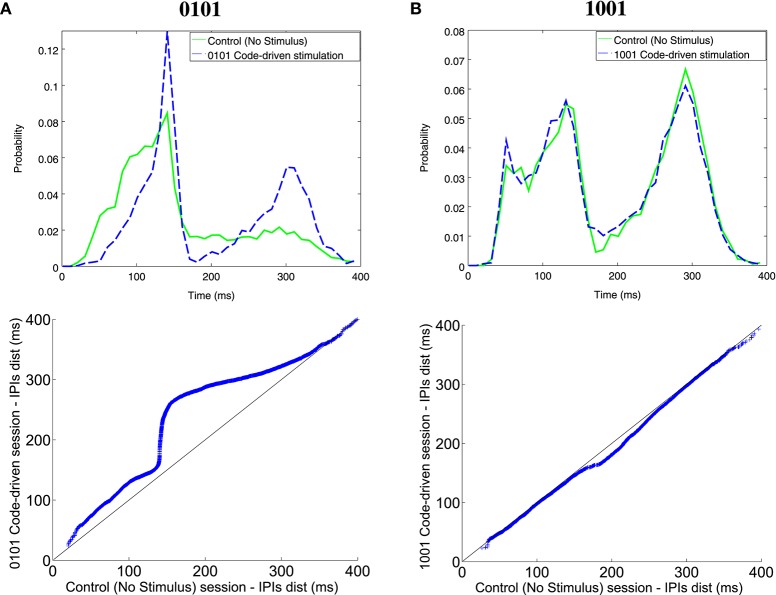
**Illustrative examples of IPI histogram and qqplots for different codes (0101, A; 1001, B)**. The IPI histograms (top) represents IPIs discharged during the control session (solid line) and the code-driven stimulation session (dashed line). IPIs, represented on the X axis, were in the range between 0 and 400 ms and the probability, represented in the Y axis, was normalized. Qqplots (bottom) represent IPI distribution during control session vs. IPI distribution during code-driven stimulation sessions. The black line represents the reference line y = x. See an additional example in Figure S3.

Comparing IPI distribution between closed-loop stimulation sessions, with 0101 and 1001 as triggers, and the control sessions, the fish increased the probability of firing longer IPIs when the trigger word was 0101 for all ranges (Figure 7A). This was also observed in the qqplot as the dots were above the reference line. Nonetheless, there were no changes in the electrical behavior when the trigger word was 1001 (Figure 7B) where the dots were almost aligned with the reference line. Further examples in different experiments are shown in Figure S2.

### 4.3. Influence of temporal sequences of events in information processing

**Figure 8:**
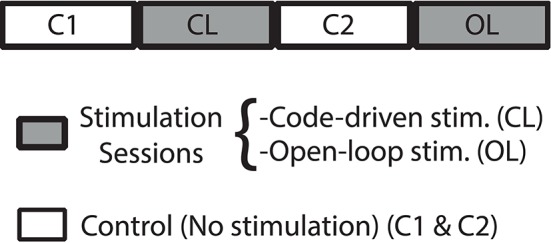
**Experimental protocol**. Closed-loop (CL) and Open-loop (OL) sessions, represented in gray, were stimulation sessions. C (control) sessions, represented in white, were sessions where no stimulation was delivered. As IPI discharges of *Gnathonemus petersii* are highly variable, we assume as our *baseline* the control session before a stimulation session.

Here, we performed 4 bit word closed-loop and open-loop, so we could compare the intrisic variability of the electrical behavioral due to random stimulation and compare it to that due to the code-driven closed-loop protocol. The experimental procedure was as follows (Figure 8):

1. Control (C1). Record fish's electric activity without stimuli.
2. Closed-loop stimulation session (CL). Code-driven closed-loop stimulation (binary word detection trigger *W*^4^ = 0101).
3. Control (C2).
4. Open-loop stimulation session (OL). Presenting the same stimuli, delay and stimulation frequency than that for the code-driven closed-loop protocol.

As IPIs of *Gnathonemus petersii* are highly variable, we assume as our *baseline* the control session before a stimulation session. We chose, as the trigger in closed-loop sessions, the word that produced more changes in the electrical behavior of the fish in the previous analyses, that is, *W*^4^ = 0101. We selected Δ*t* = 80 ms from previous characterization. We proceeded this way to compare the results showed in the previous section with changes resulting from open-loop stimulation, i.e., changes due to stimulation not depending on the system's activity.

**Figure 9:**
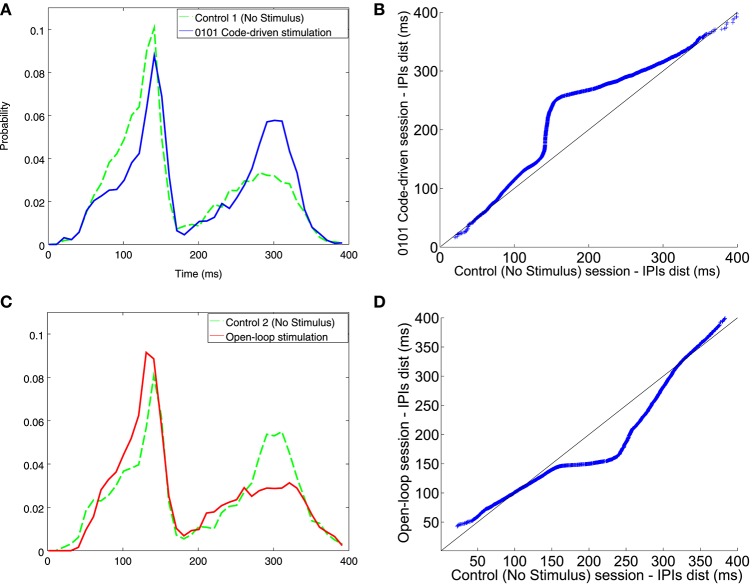
**IPI histogram and qqplots resulting from executing the code-driven and the open-loop protocols, as compared to the control session**. **(A)** IPIs discharged during the first control session (**A** dashed line) and the code-driven stimulation session (**A** solid line). **(C)** IPIs discharged during the second control session (**C** dashed line) and the open-loop stimulation session (**C** solid line). IPIs, represented on the X axis, were in the range between 0 and 400 ms and the probability, represented in the Y axis, was normalized. The qqplots represent the IPI distribution during control session vs. the IPI distribution during code-driven stimulation sessions **(B)** and open-loop sessions **(D)**. In both qqplots the black line represents the reference line y = x. See an additional example in Figure S4.

There was an increase in the probability of firing longer IPIs when stimulating using closed-loop stimulation protocol (Figure 9A), specially for IPIs from 150 to 330 ms. Oppositely, there was higher probability to fire shorter IPIs when using open-loop stimulation (Figure 9C). Another example of open-loop stimulation is depicted in Figure S3.

In the qqplot representing IPI distribution in closed-loop stimulation (Figure 9B) the points were above the reference line, thus meaning that the system fired longer IPIs. Nevertheless, when stimulating using open-loop (Figure 9D) the points lied under the reference line.

## 5. Discussion and conclusions

The study of how biological systems encode, decode, and process temporal information is a complex subject due to the high variability of temporal coding schemes, which can be sometimes superimposed. Experimental closed-loop protocols that take into account the temporal aspects of monitored signals to deliver stimulation at specific times can help in this task. The application of closed-loop stimulation protocols based on information-theoretic cost functions for selecting the stimulus (Paninski, 2005; Lewi et al., 2009) has been shown effective in several experiments (Benda et al., 2007; DiMattina and Zhang, 2011, 2014). Our closed-loop stimulation is directly triggered by the presence of the code. This implies a precise timing for the stimulation, which depends on the real-time implementation of the protocols. This approach can also reveal relevant hidden dynamics in the system, like those related with communication, learning, and system's conditioning.

We have proposed here the concept of code-driven stimulation. This controlled interaction serves to evaluate the relevance of specific sequences of events in the coding scheme of the biological system under study. In a code-driven stimulation protocol, the system is monitored, and a stimulus is delivered whenever a specific temporal code is detected in the observed signal. To implement this closed-loop approach signals are digitized to a binary sequence and analyzed in real time. The significance of a code can be evaluated as function of how the stimuli changes the response of the system to the code-driven stimulation. The insight from the code-driven stimulation protocol is enhanced by the comparison with the open-loop protocol.

This new methodology was implemented in a real-time toolbox. For validation, it was applied to the study of electric signaling in weakly electric fish. This toolbox can be used to implement both open-loop and closed-loop protocols for system characterization. The toolbox works with hard real-time constraints, as it runs under the RTAI (https://www.rtai.org/) linux extension.

Preliminary results applying this methodology in weakly electric fish showed that code-driven stimulation based on temporal sequences can modify the fish electrical behavior in a different way than when stimulation is guided by simple pulses.

The observations presented here raise the hypothesis that even a minimal sequence has relevance to the system. In addition, comparing stimulation with different words we observe that changes occur only for specific words. For instance, stimulation triggered by *W* = 1010 led to changes in the system that did not occur with stimulation triggered by *W* = 1001.

Interestingly, after a 0101 code-driven stimulation (Figure 9), similar distribution of IPIs between the closed loop session and the second control session were observed. In this case, closed-loop stimulation might have changed the firing pattern and this effect might have lasted until the second control session. Larger resting periods between sessions could discard this possibility. However, closed-loop stimulation still affects the system in a distinct manner, as open-loop stimulation neither increases nor maintains the probability of firing larger IPIs.

Finally, the comparison with open-loop stimulation emphasizes that there is a difference between stimulating after a predefined word and stimulating after a random sequence of events. One can think that different activity patterns provide information about the states in which the system can be found. These results evidence the importance of taking into account temporal patterns of events to study biological information processing.

Our methodology is open to a wide range of experimental settings. We have selected simple validation experiments but, for instance, several codes could be analyzed at once associating different triggers with different stimulations. Regarding binary digitization, we have followed a maximum entropy criterion to address the selection of bin times, but the method allows for other options. The characterization software included also implements entropy correction for the sampling bias problem in spike train information measures (Panzeri et al., 2007). The modular design of our toolbox allows to use it in conjunction with other software as, for example, PyEntropy, R Entropy, MATLAB Spike Train Analysis Toolbox, etc.

It is important to note that the criterion of maximizing entropy favors dense codes. This is appropriate in our example, as using shorter time bins would lead to sparser codes and an extremely low probability of matching an exact code. Nevertheless, it is possible to increase the temporal resolution of the method using sparser codes in conjunction with a similarity measure for triggering the stimulation. A positive strategy for measuring similarity between spike trains is to define a metric space considering pulses and IPIs duration as in Victor and Purpura (1996, 1997). It can also be used to reveal appropriate resolution for analyzing spike trains. Another approach is to use a neural network for classification of temporal patterns in spike trains in order to trigger the stimulation (Kjaer et al., 1994; Middlebrooks et al., 1994). Such method can even surmount the temporal resolution problem, although it makes more difficult for the researchers to draw conclusions about the underlying temporal code. As another counterpart, its incremented complexity hinders a real-time implementation, hence to stimulate in closed-loop. Therefore, these possible approaches must be studied in more detail.

It is important to emphasize that the applicability of the concept of code-driven stimulation is wider than that of the specific example illustrated here in the context of fish electrical signaling. In particular, the binary digitization protocols and parameters chosen in this example can be easily adapted and/or expanded for other biological systems. Furthermore, code-driven stimulation can be generalized beyond digital encoding using tools from symbolic dynamics to represent information in biological signals as sequential events whose timing can be naturally stretched or compressed in time. Correspondingly, delivered stimuli can also be designed to contain temporal structure meaningful for each specific application.

Finally, the code-driven stimulation concept also allows for a closed-loop selection of the stimulus triggering code. Of course, this increases the complexity of the experimental design but automatizes the code and the associated parameter selection, i.e., by providing a performance measurement for this characterization process.

## 6. Human search and animal research

Permission of the ethics committee of Universidad Autónoma de Madrid was obtained (TIN2012-30883). All experiments were noninvasive behavioral trials: fish were stimulated using weak electric pulses with the same amplitude as their natural electrical activity. All animals behaved normally after the experiments.

## Author contributions

Conceived and designed the experiments: AL, FD. Performed the experiments: AL. Analyzed the data: AL, CF, RP, PV, FD. Wrote the paper: AL, CF, RP, PV, FD.

## Funding

This work was funded by Spanish projects of Ministerio de Economía y Competitividad/FEDER TIN-2010-19607, TIN2014-54580-R, TIN-2012-30883, DPI2015-65833-P (http://www.mineco.gob.es/), ONRG grant N62909-14-1-N279, Brazilian Agency of Conselho Nacional de Desenvolvimento Científico e Tecnológico (http://www.cnpq.br/) and Fundação de Amparo à Pesquisa do Estado de São Paulo (www.fapesp.br). The funders had no role in study design, data collection and analysis, decision to publish, or preparation of the manuscript.

### Conflict of interest statement

The authors declare that the research was conducted in the absence of any commercial or financial relationships that could be construed as a potential conflict of interest.

## Footnotes

^1^See http://code.google.com/p/pyentropy/

^2^See http://www.strimmerlab.org/software/entropy/index.html

^3^See https://github.com/iahncajigas/nSTAT
