# Supplementary material for: Temporal Code-Driven Stimulation: Definition and Application to Electric Fish Signaling
Source: Front Neuroinform. 2016 Oct 6;10:41. doi: 10.3389/fninf.2016.00041 (PMC5052257; doi:10.3389/fninf.2016.00041)
Supplement: Supplementary file 1 [file Presentation1.PDF]

Supplementary Material: Temporal code-driven stimulation: definition and application to electrocommunication.

Angel Lareo*, Caroline Garcia Forlim, Reynaldo D Pinto, Pablo Varona and Francisco de Borja Rodriguez*

*Correspondence:
Angel Lareo, Francisco de Borja Rodriguez
angel.lareo@uam.es
f.rodriguez@uam.es

1 PSEUDO-CODE OF THE REAL-TIME PROTOCOLS

Here we present the pseudo-code of the stimulation protocols described in the paper. These protocols are the core of the presented methodology: Temporal code-driven stimulation. These protocols operate as a periodic task in real-time, executed once per interval.

```
//Code-driven Stimulation

while(running)
    t = getTime()
    V = getValue()

    //New Bin
    if (t >= binInit + binTime)
        bit[n]=0
        binInit = t
        n++

    //Detect spike and store time
    if (V > Threshold)
        bit[n] = 1
        eventTime = t

    //Detect Word
    if (wordDetected(bit[n-wordLength:n]))
        stimFlag = true

    //Check time to stimulate: Stimulate?
    if ((stimFlag==true) AND
          (t = eventTime + delay))
        STIMULUS()
        stimFlag = false
```

```
//Open-loop Stimulation

while (running)
    t = getTime()
    V = getValue()

    //Detect spike and store time
    if (V > Threshold)
        eventTime = t

    //New window
    if (t >= winInit + winTime)
            //New randomTime:
        randomTime = random(0, winTime)
        winInit = t

    //Out of randomTime
    if (t >= winInit + randomTime)
        stimFlag=true;

    //Check time to stimulate: Stimulate?
    if (stimFlag AND
        (t = spkTime + delay))
      STIMULUS();
```

The protocols have to keep track of the digitization window, the current bin and the previous bits among executions. This is accomplished in our implementation using static variables. Nevertheless, for the sake of a better understanding of the tasks performed by the protocols, here we provided a sequential pseudo-code. It represents the tasks executed in each real-time interval, whose workflow is depicted in Fig. 3 in the paper, as an iteration of a while loop.

The time and the signal value are acquired in every iteration and provided to the function using the parameters *t* and *V* respectively. *STIMULUS()* is a call to a procedure responsible for delivering the stimulus, which must be specifically adapted for each application.

2 PROVIDED SOURCE CODE

The provided source code includes an implementation of the real-time protocols, an application for offline analysis of biological signals and a library providing functions to assess binary digitization.

Folder structure:

- rt_words
- PezHist
- BinWords

2.1 rt_words

This library contains the functions that implement the real-time stimulation protocols described in the paper and in the previous section. These functions are implemented in ANSI-C. In order to be used, they have to be integrated into a real time system as a periodic task.

The scheduling of this system must guarantee that real-time tasks achieve their timing deadlines working at a high temporal resolution, with a reliable update rate of 10 kHz at least. The real-time task must poll periodically. It is responsible for obtaining the precise time (*time*) and the value of the monitored signal from the DAQ (*currentV*) and for doing the function calling.

We declared a model function which serves as an interface to run the real-time protocols (described in the previous section). The periodic real-time task must call *model(currentV,time)* at each interval.

The declaration of the model function is as follows:

```
double model(double currentV, double time)
```

The parameters for the protocols have to be previously selected filling the structure words_shm. The word size can be selected by filling the field *wordSize*.

Declaration of the structure words_shm:

```
typedef struct
{
    char activated[3];   //[0] - WordsDetector    - Temporal code-driven stimulation
                         //[1] - WordsHistogram   - Generate histograms
                         //[2] - aleatModel       - Open-loop stimulation

    double threshold;
    double bitTime;      //binTime
```

```
    int wordSize;          //word length (number of bits)
    char word[MAX_BITS_WORD]; //trigger
    int numWords;          //number of words
    double windowTime;     //window time
    double refractTime;
        int hist[MAX_WORDS]; //histogram: Stores #word
        unsigned char histSem;

    int channel_in;        //monitored channel

    double randomPeriod;//windowTime for Open-Loop stimulation
    double minDelay;       //minDelay
    double maxDelay;       //maxDelay
} WORDS_SHM;
```

Regarding stimulation, the functions call a procedure named *trigger_function* which is responsible for delivering the stimulus.

2.2 BioHist

This software performs binary digitization of a given biological signal in an offline way. This task is the characterization depicted in Fig. 1 in the paper, previous to the code-driven stimulation loop. For a given set of digitization times (Δt) and word lengths (L) it calculates all possible histograms, the entropy associated to each histogram, the bias estimation and the corrected entropy.

The signal has to be previously recorded in a file with two column vectors and n rows. The first column has to store the time in which the sampling takes place, the second column has to store the signal value at that precise moment. Each row must be a sample.

It *PezHist* generates an HDF5 file containing histograms of words and entropy results.

This is a basic characterization tool, provided to ease the selection of appropriate parameter values. Other software tools for information-theoretic analysis of neural data can be used independently or in conjunction with it. For instance, some of these tools are PyEntropy [1], R Entropy [2] or MATLAB Spike Train Analysis Toolbox [3].

Requirements:

- C++11
- Boost>=1.53
- HDF5
- YamlCpp

Compile:

```
$ cmake .
```

[1] See http://code.google.com/p/pyentropy/

[2] See http://www.strimmerlab.org/software/entropy/index.html

[3] See https://github.com/iahncajigas/nSTAT

```
$ make
```

Usage:

```
$ ./bin/PezHist config.yaml
```

Remember to fill config.yaml. An example file is provided.

2.2.1 Working with the HDF5 output file

We provide some examples of how to work with the output file using hdf5 tools.

Show all data in the output file:

```
$ h5dump -H <file.h5>
```

Print entropy values as a matrix:

```
$ h5dump -d Entropy/Data <file.h5>
```

Dump entropy to plain text:

```
$ h5totxt -d Entropy/Data -s <separator> -o <txt file> <file.h5>
```

2.3 BinWords

A library for binary digitization written in ANSI-C. It contains the subroutines that we used in order to store and manage the binary words.

Compile:

```
$ cmake .
$ make
```

2.3.1 Application Programming Interface (API)

```
int wbInit(WordsBuffer* wb, int length, int maxWords);
```

wbInit constructs a new WordsBuffer. This buffer keeps track of a sequence of bits with length equal to the *length* parameter. It can also store binary words as integer values.

```
int wbBitInsert(WordsBuffer* wb, char bit);
```

wbBitInsert inserts a new bit in the buffer.

```
int wbStoreWord (WordsBuffer *wb);
```

wbStoreWord stores the binary sequence of bits in the buffer as an integer value.

```
int wbCheckWordMatch(WordsBuffer *wb, char *word);
```

wbCheckWordMatch checks if the binary word provided as a parameter (*word*) matches with the binary sequence of bits stored in the buffer.

```
void wbCreateHistogram (WordsBuffer* wb, int* results, int numWords);
```

wbCreateHistogram fills in the array *results* with the number of occurrences of each possible word.

3 CODE-DRIVEN STIMULATION VS PULSE-EVENT TRIGGERED STIMULATION: COMPARISON WITH CONTROL DATA

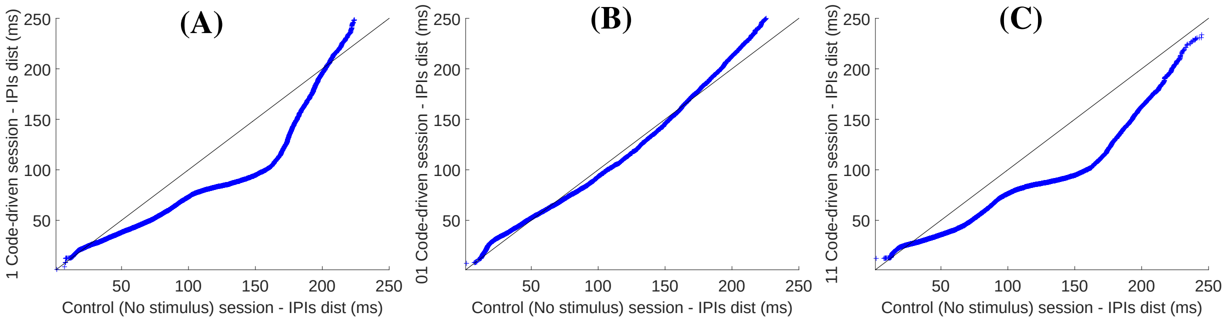

Figure S1. Complementary IPI histogram and qqplots resulting from experiments addressing temporal coding influence with minimal codes, including IPIs from control session (Fig. 6 in the paper). The IPI histogram represents the distribution of IPIs discharged during code-driven stimulation sessions with different words triggering the stimulation (1 - solid line; 01 - dashed line; 11 - dotted line; Control - black solid line). IPIs, represented on the X axis, were in the range between 0 ms and 250 ms and the probability, represented in the Y axis, was normalized. The qqplots represented IPI distributions during control sessions stimulation sessions versus code-driven stimulation sessions (1 A; 01 B; 11 C). X=Y is the reference line.

4 ADDITIONAL VALIDATION EXAMPLES OF TEMPORAL CODE-DRIVEN STIMULATION

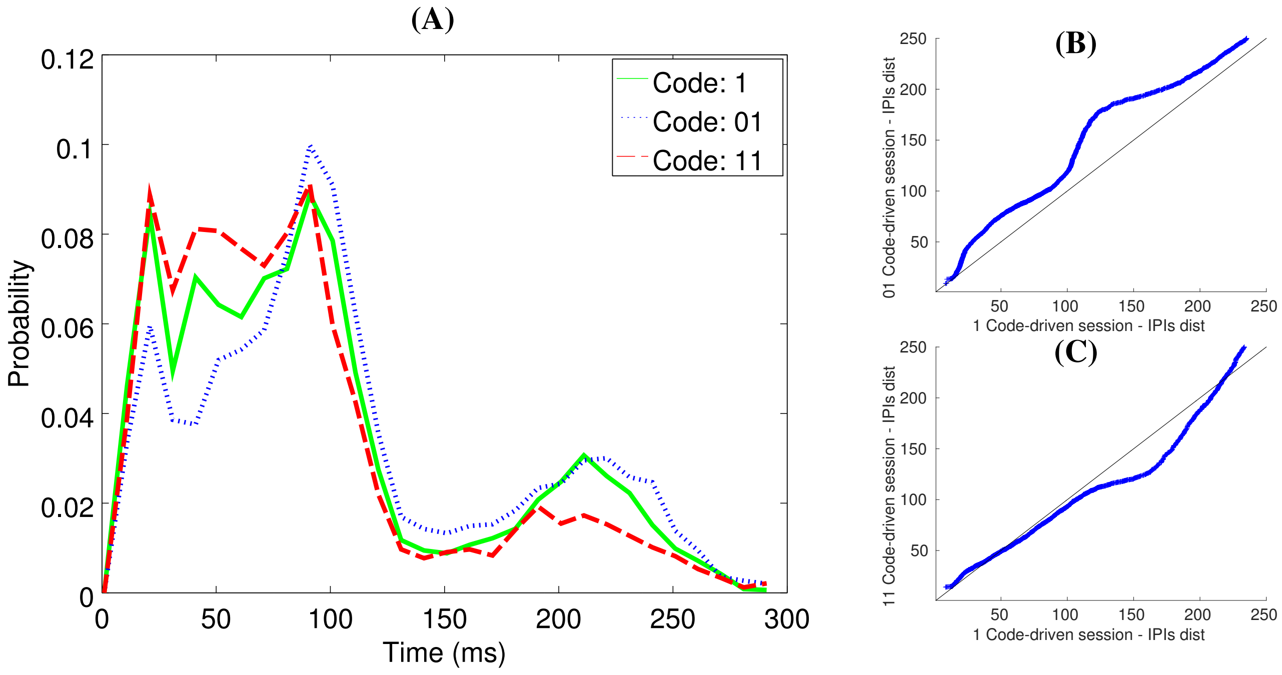

Figure S2. Illustrative example of IPI histogram and qqplots resulting from additional validation experiments addressing temporal coding influence with minimal codes. As in Fig. 6 in the paper, the IPI histogram (A) represents the distribution of IPIs discharged during code-driven stimulation sessions with different words triggering the stimulation ('1' solid line; '01' dashed line; '11' dotted line). IPIs, represented on the X axis, were in the range between 0 ms and 300 ms and the probability, represented in the Y axis, was normalized. The qqplots represented IPI distributions during pulse event ('1') stimulation sessions versus 2-bit code-driven stimulation sessions ('01' B; '11' C). X=Y is the reference line. In the qqplot that represented IPIs during the stimulation session using pulses versus IPIs during the '01' stimulation session (B) most of the points were above the reference line, thus indicating that IPIs discharged during the '01'-session were larger than those from the '1'-session. In the qqplot that represents IPIs during stimulation session using pulses versus IPIs during 11 stimulation session (C), most of the points were below the reference black line, thus indicating that IPIs discharged during the '11'-session were shorter than those for the '1'-session, particularly in the range between 100 ms and 240 ms.

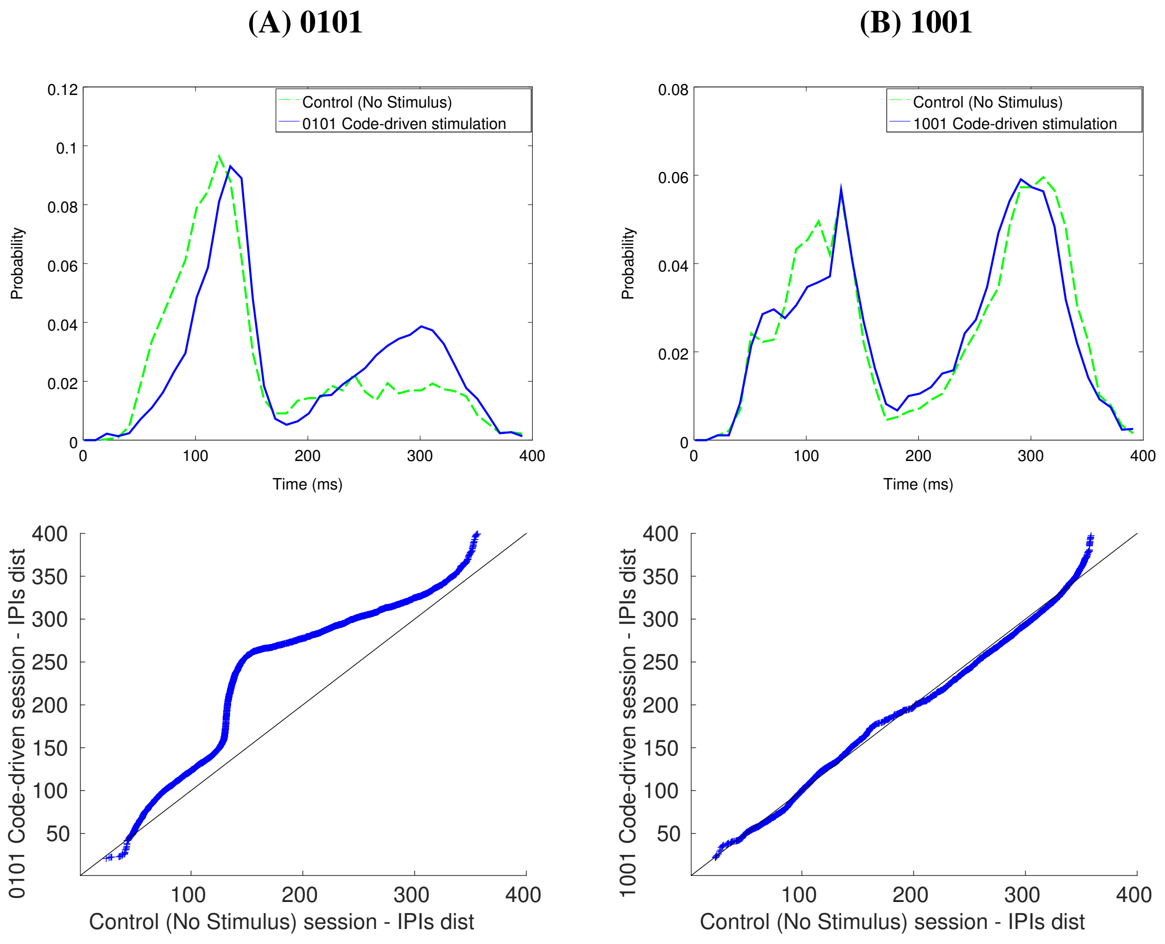

Figure S3. Illustrative example of IPI histogram and qqplot for codes '1001' and '0101' in another validation experiment. This figure shows additional experiments similar to those depicted in Fig. 7 in the paper. The IPI histogram (left) represents IPIs discharged during the control session (solid line) and the code-driven stimulation session (dashed line). IPIs, represented on the X axis, were in the range between 0 ms and 400 ms and the probability, represented in the Y axis, was normalized. Qqplot (right) represents IPI distribution during control session versus IPI distribution during code-driven stimulation sessions. The black line represents the reference line y=x. When the trigger word was '0101', the fish increased the probability of firing longer IPIs. Nevertheless, the fish did not change its IPI distribution when stimulated with '1001' trigger word. This behavior is similar to that in Fig. 7.

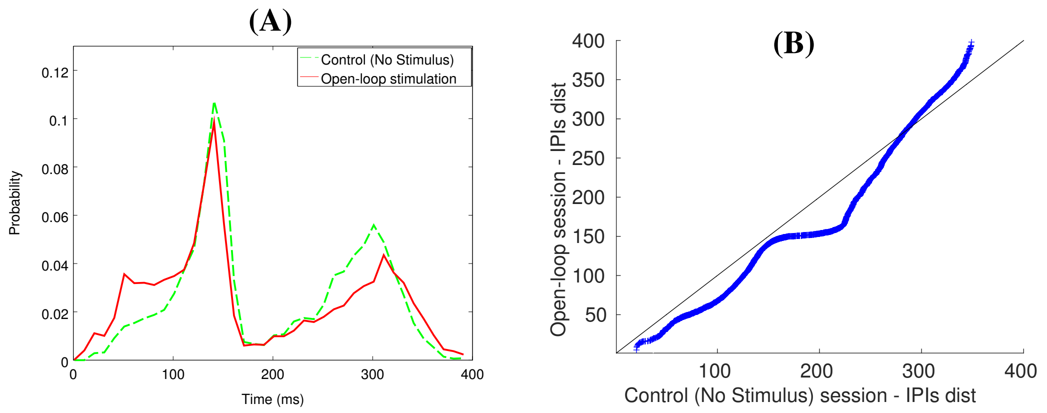

Figure S4. Illustrative example of open-loop stimulation, which complements Fig. 9 in the paper. (A) IPIs discharged during the control session (A - dashed line) and the open-loop stimulation session (A - solid line). IPIs, represented on the X axis, were in the range between 0 ms and 400 ms and the probability, represented in the Y axis, was normalized. The qqplots represent the IPI distribution during control session versus the IPI distribution during open-loop sessions (D). The black line represents the reference line y=x. Similarly to Fig. 9, when using open-loop stimulation the fish increased the probability of firing shorter IPIs. Oppositely, closed-loop stimulation using the triggering word '0101' increased the probability of firing longer IPIs, as depicted in Fig. S2-A, Fig. 8-A and Fig 9-A,B in the paper.
