# Supplementary figures and images for: Temporal Code-Driven Stimulation: Definition and Application to Electric Fish Signaling
Source: Front Neuroinform. 2016 Oct 6;10:41. doi: 10.3389/fninf.2016.00041 (PMC5052257; doi:10.3389/fninf.2016.00041)

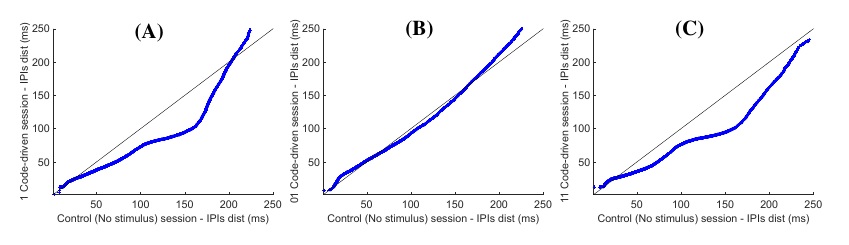

Supplement: Supplementary file 3 [file Image1.JPEG]

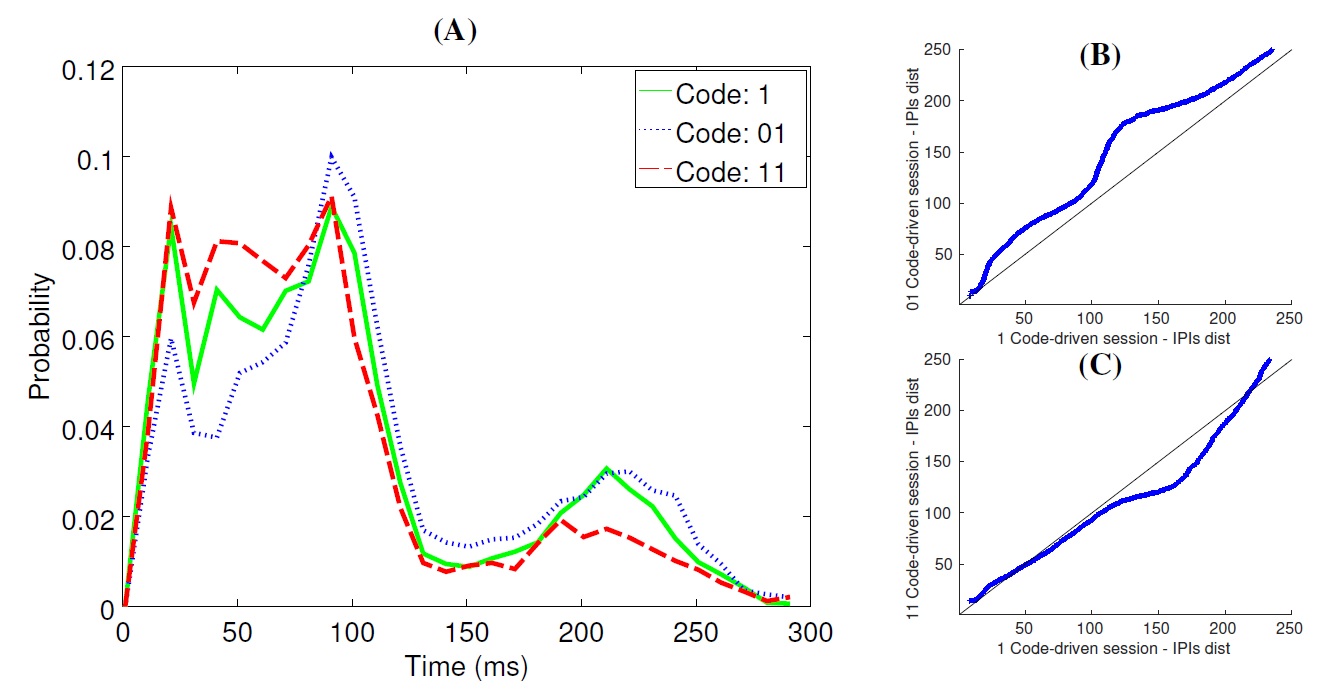

Supplement: Supplementary file 4 [file Image2.JPEG]

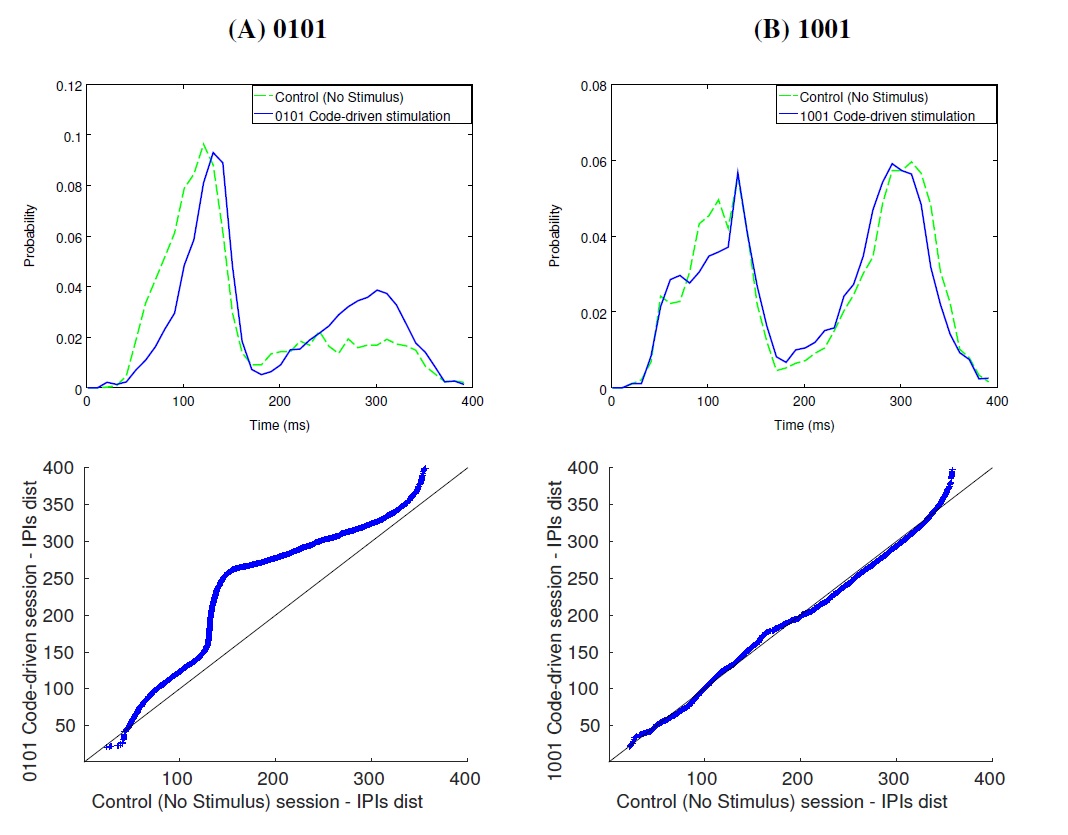

Supplement: Supplementary file 5 [file Image3.JPEG]

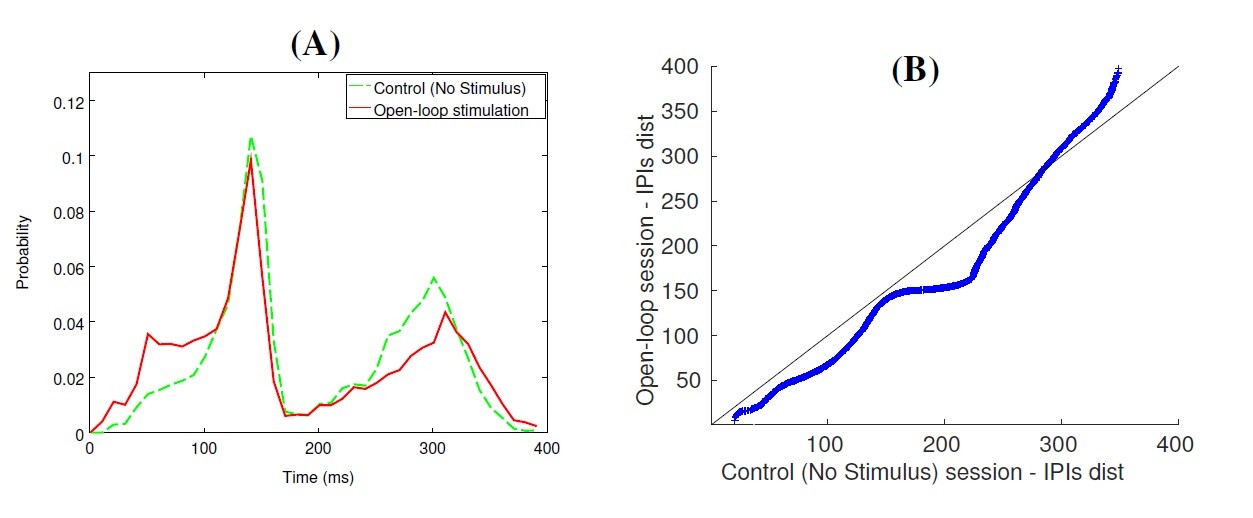

Supplement: Supplementary file 6 [file Image4.JPEG]
